# Temperature and salinity preferences of endangered Delta Smelt (*Hypomesus transpacificus*, Actinopterygii, Osmeridae)

**DOI:** 10.1038/s41598-022-20934-w

**Published:** 2022-10-03

**Authors:** Tien-Chieh Hung, Bruce G. Hammock, Marade Sandford, Marie Stillway, Michael Park, Joan C. Lindberg, Swee J. Teh

**Affiliations:** 1 Fish Conservation and Culture Laboratory, Department of Biological and Agricultural Engineering, University of California, Davis, CA 95616 USA; 2 Aquatic Health Program, Veterinary Medicine: Anatomy, Physiology, and Cell Biology, University of California, Davis, CA 95616 USA

**Keywords:** Animal behaviour, Behavioural ecology

## Abstract

Temperature and salinity often define the distributions of aquatic organisms. This is at least partially true for Delta Smelt, an imperiled species endemic to the upper San Francisco Estuary. While much is known about the tolerances and distribution of Delta Smelt in relation to these parameters, little is known regarding the temperature and salinity preferences of the species. Therefore, the temperature and salinity preferences of sub-adult Delta Smelt were investigated across a wide range of thermal (8–28 °C) and salinity (0–23 ppt) conditions. Replicates of ten fish were allowed to swim between two circular chambers with different temperature or salinity, and the distribution of fish between the chambers was recorded. We found that Delta Smelt showed no temperature preference below 15 °C, a modest aversion to the warmer tank from 15 to 28 °C, and a strong aversion to the warmer tank with elevated mortality at temperatures above 28 °C. Delta Smelt also preferred lower salinities, and this preference became more pronounced as salinity increased toward 23 ppt. These results indicate that Delta Smelt can tolerate high temperatures and salinities for a short time, and that their preferences for lower temperature and salinity strengthens as these variables increase.

## Introduction

Delta Smelt, *Hypomesus transpacificus* McAllister, 1963, is a small, silvery fish endemic to the upper San Francisco Estuary (SFE), where water from the Pacific Ocean mixes with freshwater from the Sacramento and San Joaquin rivers. Delta Smelt were once abundant in the SFE, inhabiting the freshwater Delta and brackish regions such as Suisun Bay^1^. Today, abundance of Delta Smelt in the wild is extremely low^2^, and the species is listed as threatened federally^3^ and as endangered under the California Endangered Species Act^4^. Several potentially interacting factors are thought to have contributed to the decline in Delta Smelt, including a loss of food resources, decreased turbidity, changing hydrodynamics associated with freshwater exports, loss of tidal wetlands, and an extended recent drought^5–10^. The recent drought from 2012 to 2016 was followed by one of the wettest years on record for California (2017), which provided hope that a natural recovery would occur, similar to that observed in 2011. However, numbers of Delta Smelt did not increase substantially despite high outflow^10^, perhaps due to relatively high water temperatures in 2017^11^. Recent abundance trends strongly suggest that Delta Smelt are nearly extinct in the wild, and most recent surveys to locate Delta Smelt have failed to detect the species^12,13^. Then, from December 2021 through February 2022, more than fifty thousand hatchery-raised adult Delta Smelt were reintroduced to the SFE to supplement the wild population, a first for the species. Thus, it is currently unclear whether the wild population is largely of hatchery or wild origin.

Increased temperature and salinity are considered two primary mechanisms by which droughts reduce Delta Smelt range and abundance. Low outflow during droughts shifts the salinity field landward, reducing the quantity and quality of habitat for Delta Smelt^8^. Decreased freshwater outflow is also associated with increased water temperature in the Delta^14,15^. Climate change is expected to exacerbate these effects^16^, with projected increases in temperature and salinity exerting direct effects on physiological and fitness parameters of Delta Smelt and increasing extinction risk (i.e., energy allocation, growth, reproduction)^9,10,17^. For example, elevated water temperatures are associated with declines in otolith increment and condition factor of Delta Smelt collected from the wild^18,19^.

Considerable research has been conducted on the salinity range of Delta Smelt in the environment and their tolerances in the laboratory. Delta Smelt have been found at salinities up to 18 ppt in the wild^20^ and can survive salinities up to 34 ppt in the lab (96-h adult survival 81.5%)^21^. However, Delta Smelt are historically most abundant^22–24^ and stomach fullness peaks^25^ in the freshwater to low salinity zone (LSZ) of the estuary (0.5–6 ppt). The LSZ is a salinity range that moves seaward and landward with freshwater and tidal flows and is centered at a salinity of about 2 ppt^23^.

Critical thermal methodology is used to quantify a maximum temperature at which fish are unable to escape unfavorable conditions that would eventually result in their mortality (i.e., CT_max_)^26,27^. Plasticity in high temperature tolerance with changes in acclimation temperatures is commonly observed in fishes, including Inland Silverside (*Menidia beryllina* Cope, 1867), Largemouth Bass (*Micropterus salmoides* Lacépède, 1802), and Delta Smelt^28^. For example, several studies have demonstrated that a warm acclimation temperature can increase a species’ CT_max_ relative to optimum acclimation temperature^27^. For Delta Smelt, Swanson et al. reported a critical thermal maximum of 25.4 °C when acclimated at 17°C^29^. Komoroske et al. acclimated juvenile Delta Smelt at 18.7 °C, which resulted in a lethal CT_max_ of 27–28 °C^21^. Davis et al. recorded some of the highest acute upper temperature tolerance limits for juvenile Delta Smelt after 20 °C acclimation, with a CT_max_ of 29.7 °C ± 0.2 (mean ± SE), with 25% of individuals tolerating temperatures of 30.0–30.7 °C^28^.

Elevated water temperatures increase the metabolic demand of ectotherms like Delta Smelt, or they may directly cause mortality if the CT_max_ is approached^26,28^. Although ectotherms can generally compensate for elevated metabolic demand by increasing foraging, this may be difficult for Delta Smelt given the low availability of prey in the SFE^25,30,31^. Although Delta Smelt have been found in the wild at temperatures from 6 to 25 °C, they are most often found in areas with temperatures < 22 °C^20,28,32–34^. Unfavorably high temperatures are increasingly characteristic of much of the Delta in the summer and are associated with the absence of Delta Smelt from the central and south Delta^2^.

While much is known regarding Delta Smelt temperature and salinity tolerances and distribution in the environment, little is known regarding the temperature and salinity preferences of the species. Distribution data of Delta Smelt in the wild is based on a multitude of variables likely to also influence distribution, such as prey availability, turbidity, predators, and competitors, making it difficult to infer preferences from distribution data. For example, high temperatures may cause individuals to emigrate from a habitat due to a preference for lower temperatures, or temperature may interact with other ecological variables such as prey scarcity. However, temperature and salinity are generally considered as major drivers since most California native fish species were identified as highly vulnerable to warming temperatures^35^, and Delta Smelt belongs to a family of fish that does not extend too far south of the San Francisco Estuary along the eastern Pacific Coast^20^. In addition, the majority of Delta Smelt population is semi-anadromous^36^ with movement highly associated with turbidity and salinity gradients in water^32,37^. Moreover, most laboratory-based tolerance studies do not offer individuals a choice, whereas wild animals typically can choose their habitat. Preference data can be especially important for understanding why a population might become extirpated during a drought, which could be caused by a behavioral response, acute mortality, or an interaction between abiotic and ecological variables (e.g., prey, predators, competitors, and disease).

Inferring temperature and salinity preferences of aquatic organisms from behavioral experiments is well-established. For example, Hirvonen et al. used a y-maze fluvarium where Arctic Charr (*Salvelinus alpinus* Linnaeus, 1758) could choose between control water or stimulus water with fish odor^38^. A y-maze was also used by Correia et al. to explore the influence of turbidity and prey chemical cues on the foraging behavior of Red Swamp Crayfish (*Procambarus clarkii* Girard, 1852)^39^. Nay et al. used a shuttlebox system to test for an interaction between habitat complexity and temperature in a common coral reef fish^40^. Here, we investigated the preferential behavioral response of sub-adult Delta Smelt to thermal and salinity conditions loosely based on critical thermal methodology (CTM). Preference experiments were conducted across a wide range of temperatures and salinities using a shuttlebox system (Loligo Systems, https://www.loligosystems.com/core-shuttle-box-system). The shuttlebox system consisted of two tanks connected by a narrow passage, allowing individuals to select the tank with the most suitable conditions. Our hypotheses were that (1) Delta Smelt will avoid the environment with higher temperature or salinity after that parameter reaches a certain level, but only below the lethal level at which a choice is no longer possible; (2) temperature and salinity preferences will strengthen as the water becomes increasingly warm or saline, respectively; and (3) differences in acclimation temperature will alter temperature preference, with higher acclimation temperature lessening any preference for cool water.

## Materials and methods

### Fish collection and care

Cultured Delta Smelt at juvenile to sub-adult stages (38–69 mm in fork length; mean ± SD: 55.7 ± 6.7 mm) were transported from the UC Davis Fish Conservation and Culture Laboratory (FCCL) in Byron, CA, to the UC Davis Aquatic Health Program in Davis, CA. Fish were held in a recirculating, biofiltered system at 16 °C, using reconstituted water prepared according to the United States Environmental Protection Agency guidelines^41^. Water quality was kept within the physiological ranges of the Delta Smelt during pre-experimental culture (total alkalinity of 80 mg/L, hardness of 102.5 mg/L, electrical conductivity of 530 µmhos/cm, salinity of 0.4 ppt, and pH of 7.6). The fish were fed four times a day at 3% body mass per day with a mixture of formulated 4/6 NRD diet (INVE Aquaculture, Salt Lake City, Utah) and 370 Hikari plankton food (By-Rite Pet Supply, Hayward, California)^42^. Uneaten food was removed at the end of each one hour feeding by siphoning the bottom of the tank. A 25% water change was performed every day. Periodic lighting was provided (14-h light: 10-h dark photoperiod) with fluorescent lighting.

Delta Smelt (n = 120) were then transferred to and held in three 300-L circular tanks and acclimated for one week prior to the preference trials (one 300-L tank for each acclimation/trial procedure). For the temperature preference trials, fish were held in acclimation tanks at either 14 °C (‘cold’) or 17 °C (‘warm’), to determine how acclimation temperature affected preference values. For the salinity preference trials, Delta Smelt were slowly acclimated from 16 °C (culture temperature at the FCCL) to 20 °C over the course of one week in 0.4 ppt reconstituted water in the third 300-L tank. Fish used for the experiment were euthanized using buffered tricaine methanesulfonate (MS-222, 500 mg L^-1^) at the end of the study.

### Testing system configuration

The shuttlebox testing system consisted of two connected circular chambers, two mixing towers, and two water resource tanks. A narrow passageway connected one chamber with the other, allowing fish to swim between the chambers. Water recirculated among the chambers and the mixing towers, as shown in Fig. S1. The working volume of each circular chamber was 120.5 L (100 cm in diameter and 15 cm in depth), and the total volume of water in the recirculating system was 270 L. Water was aerated with an air stone in each mixing tower to provide adequate dissolved oxygen levels. Due to the sensitivity of Delta Smelt to light^42^, only four infrared lights (Infrared basking spot lamp PT2142-R20/75 W, Exo Terra’s, MA) were used to provide indirect illumination throughout the study (lights directed towards the ground underneath the circular chambers). A digital video camera (Pentax TV Lens 4.2 mm 1:1.6) was mounted overhead while the movement of fish was recorded by the CamStudio Recorder (version 2.7.2, http://camstudio.org/) at a frequency of 2 frames per minute. Images were analyzed using the ShuttleSoft behavior software (version 2.6.3, http://www.loligosystems.com/software).

### Flow and temperature simulations and validation

The flow patterns and temperature distribution inside the shuttlebox testing system were numerically simulated to understand the degree of mixing between the two chambers. The configuration of the system and the computational grids were built using Gambit (version 2.0.4, ANSYS Inc.). The grids representing the flow domain were created by partitioning the volume of the system into many small control volumes after the model was created. The numerical solutions to the governing equations for fluid flow in each of the grids were solved with a software package, Fluent (version 6.3.26, http://www.ansys.com/products/fluids/ansys-fluent). Fluent uses a finite volume method to approximate the solution to the governing equations for fluid and particle flows in each of the control volumes. The power law was used to solve the momentum discretization. The calculated results were displayed graphically. The simulations of temperature distribution were validated using HOBO pendant temperature/light probes (Onset, MA), a thermocouple reader with six probes (Tegam Inc., OH), and flow patterns were validated with the addition of food dye.

### Experimental design

There is some evidence that Delta Smelt exhibit shoaling behavior^43^, so three replicates of ten fish per replicate were used during each preference trial to test their volitional movements in the shuttlebox apparatus. Most similar studies have examined the behavioral responses of a single fish to different levels of an environmental stimulus^44–47^, rather than the multiple fish used here.

Delta Smelt were not fed during the preference trials—which lasted up to six days—to avoid introducing any factors that could elicit a preference for a particular side of the shuttlebox. Hammock et al. found that six days of fasting at 16 °C did not influence most biomarkers of nutritional stress in Delta Smelt (with hepatosomatic index as an exception)^48^; therefore, we do not expect that fasting would affect preferences during the experimental trials. Nevertheless, we included ‘time since last feeding’ as a covariable in our preliminary analyses. We found that it was not a significant predictor for either temperature or salinity preference; therefore, it was not included in the model comparisons presented here.

### Temperature preference trials

Temperature preference trials were 6-days in length and included temperatures ranging from 8 to 28 °C at a salinity of 0.4 ppt. Temperature target pairs (i.e., the nominal water temperatures of the two circular tanks of the shuttlebox system) included the following: [8, 10], [10, 12], [12, 14], [14, 16], [16, 18], [18, 20], [20, 22], [22, 24], [24, 26], and [26, 28] °C (± 0.5 °C). Ten fish were arbitrarily selected from either the ‘cold’ or ‘warm’ acclimation tanks and placed into the shuttlebox system for an additional six-hour acclimation period (to recover from handling stress) before beginning each six-day temperature trial. For 14 °C-acclimated fish, we placed fish in the shuttlebox chambers at 12 and 14 °C and acclimated for a 6-h period; for 17 °C-acclimated fish, the 6-h acclimation period started at 14 and 16 °C. To begin the experiment, the number of fish in each tank was counted every thirty minutes over a six-hour period using a video monitor at the initial temperatures (12 and 14 °C or 14 and 16 °C). Then, we kept the cooler temperature tank at the same temperature but changed the warmer temperature tank down 4 °C to the next lower assigned pair and once temperatures had stabilized, the distribution of fish between the tanks was again recorded every 30 min for six hours. We kept lowering temperatures and collecting distribution data until we had collected data at the lowest pair, 8 and 10 °C. Temperatures were then raised, pausing at each temperature pair to collect distribution data as above, until we reached the final temperature pair, 26 and 28 °C. Once data were collected at the highest temperature pair, the fish were removed from the tanks and the process began again with 10 different fish once temperatures had stabilized at 13 and 15 °C or 15 and 17 °C. Each tank alternated being decreased or increased in 4 °C increments over the course of each of the eight 6-day trials. Thus, only one tank changed temperature at a time (4 °C increase or decrease), such that each tank alternated serving as the ‘warmer’ tank (Fig. S2). This strategy provided an incentive for the fish to swim between the chambers to stay at their preferred temperature and minimized any tank effect (i.e., an innate preference for a particular tank). Ten fish were used for each trial; three trials were performed for the fish acclimated to 14 °C and five trials for those acclimated to 17 °C. Thus, 80 fish total were used in the temperature preference experiments. The distributions of individuals for each of the 30-min periods were averaged, rounded to the nearest integer, and used as the response variable in the analysis.

### Salinity preference trials

Salinity preference trials were 20-h in length and consisted of a broad range of salinities, ranging from freshwater to 23 ppt. Salinity target pairs included the following: [0, 0.5], [3, 5], [8, 10], [13, 15], [17, 20], and [20, 23] (± 0.5 ppt). Salinity trials were tested at a temperature of 20 °C, within the range of temperatures Delta Smelt commonly inhabit in the wild^31^ during summer saltwater intrusion. Instant Ocean sea salt (Spectrum Brand Inc., USA) was used to adjust salinity. Ten fish were arbitrarily selected from the freshwater acclimation tank and placed into the shuttlebox system for an additional six-hour acclimation period before each trial to recover from handling stress. The system salinity was increased manually every three hours in a series of 5 ppt increments with a target salinity difference of 3 ppt between the two shuttlebox chambers (actual difference was 2.2 ppt, SD = 0.9 ppt). We note that the salinity differences between the tanks were smaller at lower salinities because the differences were harder to maintain. Unlike the temperature trials during which water could be recirculating in the system throughout the trial periods, water for the salinity trials needed to be discharged in order to maintain the salinities in the source tanks for adjusting the salinity in the chambers (Fig. S1). Therefore, two approaches were used to reduce the amount of reconstituted fresh and saline water needed and to make the trial possible: (1) one random chamber was assigned to be the one with a higher salinity throughout the trial period and (2) we reduced the duration of each salinity pair to three hours from the six hours used in the temperature trials. The total number of fish in each tank was counted every 15 min during the last two hours of each salinity trial once the salinity stabilized, using a video monitor after the system salinity reached the assigned level. Ten fish were used for each trial, and three trials were performed; thus, 30 fish total were used in the salinity preference experiments. The distributions of individuals for each of the 15-min periods were averaged, rounded to the nearest integer, and used as the response variable in the analysis.

### Data analysis

The temperature preference data were plotted to check for potential thresholds and nonlinearities and then analyzed using model comparison^49^. A series of five models were fit using R in which the proportion of fish in the cooler tank was the response variable and a binomial distribution of error was used^49,50^. ‘Trial’ was included as a random effect in all models to account for the repeated measurements from the same group of ten fish^49^. Data from trials with > 50% mortality were not used to fit models. We included an intercept model (null; Model 1), a model with temperature in the warmer of the two tanks as a linear effect (Model 2), temperature of the warmer tank binned into < 28 and > 28 °C (Model 3), and temperature in the warmer tank binned into < 14, 14–28, and > 28 °C (Model 4; Table 1). Of these models, Model 4 was best, so Model 5 included the three-bin temperature variable plus a parameter for acclimation temperature (14 or 17 °C; Table 1). We note that the binned temperature variables were assigned post-hoc, based on the plots of the temperature preference results, and were meant to account for the nonlinear relationship between temperature preference and water temperature apparent from the data. The models were fit in R using the ‘glmer’ command from the ‘lme4’ package^51^. The models were compared using Akaike Information Criterion corrected for small sample size (AIC_c_)^49^. Statistical significance was determined by examining 95% confidence intervals of parameter estimates.

**Table 1:** Model comparison for the temperature preference experiment. 'Temp 3bins' is a variable in which the temperature of the warmer tank was divided into three bins, low (9.1–13.2 °C), medium (14.0–28.0 °C), and high temperature (28.1–28.8 °C). 'Temp 2bins' is a variable in which the temperature of the warmer tank was divided into two bins, < 28.0 °C and > 28.0 °C. 'Temp' is the temperature in the warmer tank as a continuous variable. 'Acclimation T' is the temperature at which Delta Smelt were acclimated (14 or 17 °C).

Model #	Model	ΔAIC_c_	df	AIC_c_ wt
4	Temp 3bins	0.0	4	0.6022
5	Temp 3bins + Acclimation T	1.5	5	0.2849
3	Temp 2bins	3.9	3	0.0875
2	Temp	6.7	3	0.0212
1	Intercept	9.9	2	0.0043

*ΔAIC*_*c*_: Difference between model of interest and top-ranked model in Akaike Information Criterion Units corrected for small sample size, *df*: Degrees of freedom, and *AIC*_*c*_* wt*: Akaike weight.

The salinity preference data were also analyzed by first plotting the data to check for thresholds and nonlinearities, and then comparing a series of binomial models with a random effect for ‘trial’. The proportion of individuals in the less saline tank was the response variable. The salinity preference data appeared fairly linear, so three models were fit: an intercept model (null; Model 1), a model with salinity in the more saline tank as the predictor variable (Model 2), and a model with the difference in salinity between the two tanks as the predictor variable (Model 3). The intercept model tested whether there was a salinity preference (if the 95% CI of the model did not overlap 50%), while the model with a salinity parameter tested whether the proportion in the fresher tank changed across the salinity gradient. Model 3 was included to test whether the apparent strengthening of the preference of Delta Smelt for the fresher water (see Results) was due to an increase in the difference between the fresh and more saline tanks at higher salinities (the correlation coefficient between salinity in the higher tank and the difference in salinity between the higher and lower tanks was 0.72). Neither the top-ranked temperature nor the salinity model was over-dispersed based on tests for over-dispersion using the ‘dispersion_glmer’ command from the R package ‘blmeco’^52^.

### Ethics declarations

All the fish transportation, handling, euthanizing, and the experimental procedures were following protocols approved by UC Davis Institutional Animal Care and Use Committee (protocol #18125). The use of fish was permitted under the Federal Fish and Wildlife Permit (#TE027742-3) and California Endangered Species Act Memorandum of Understanding.

## Results

### Temperature preference trials

**Figure 1:**
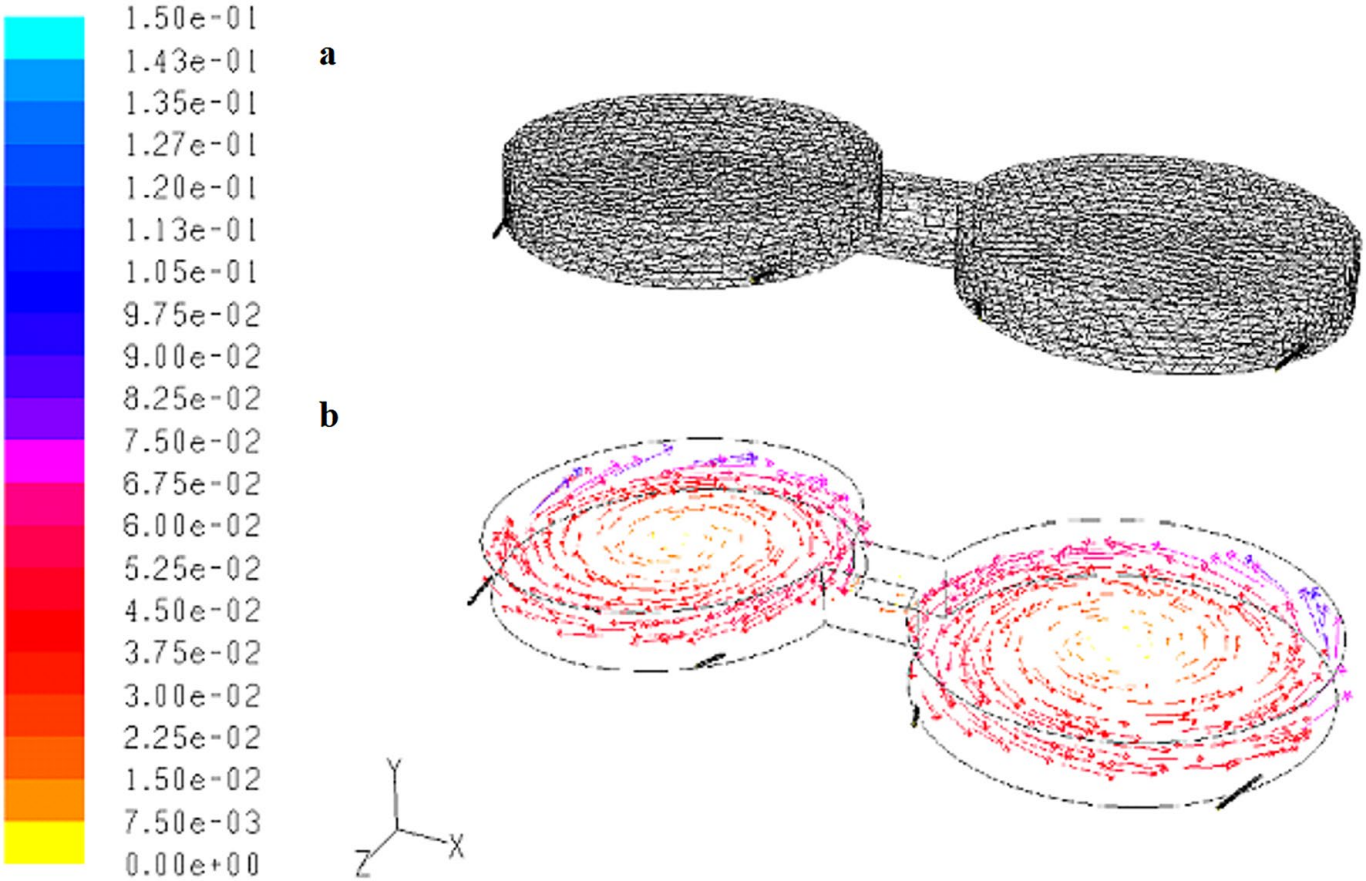
Numerical simulation of the flow patterns in the testing system. (**a**) The configuration of the model and computational grids used and (**b**) velocity vectors (m/s) at the middle of cross section of the system.

Numerical simulation demonstrated that the flow exchange between the two chambers was negligible (Fig. 1) and that temperature was uniformly distributed across each chamber. The numerical simulation demonstrates that the testing system was able to maintain a difference between the chambers (the average difference between the cooler and warmer tanks was 1.3 °C [SD = 0.5 °C]). However, ambient air temperature had a significant effect on the water surface, especially when the difference between the air and water temperature was large (Fig. S3). In addition to the numerical simulation, the flow pattern validation using food dye also showed minimal water exchange between the two chambers.

**Figure 2:**
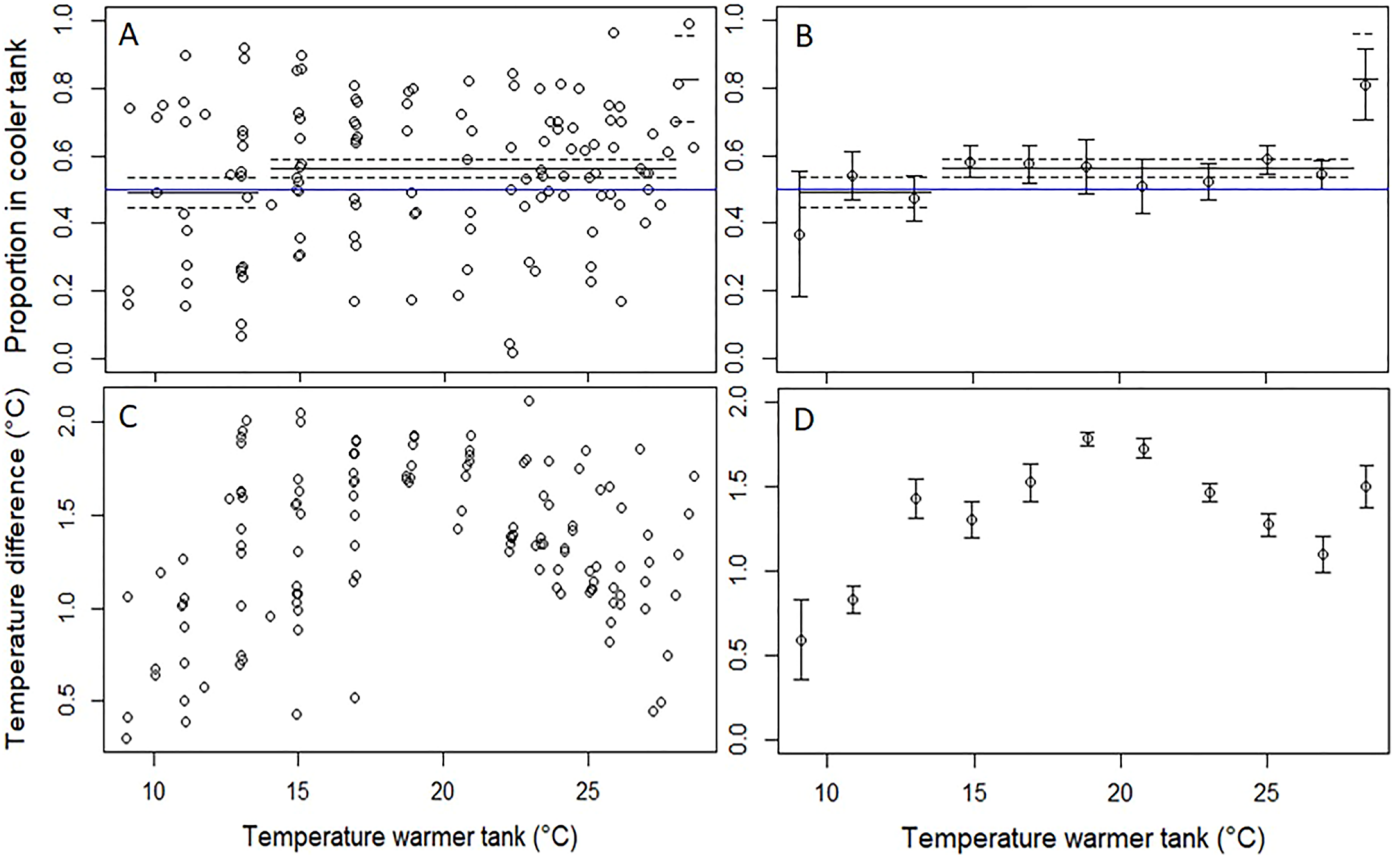
Panel (**A**) shows the proportion of sub-adult Delta Smelt in the cooler tank versus water temperature in the warmer tank. The solid black line represents the top-ranked model and the dashed lines are the 95% CI. Panel (**B**) shows the same as panel (**A**), but with means ± SE for temperature bins rather than raw data. Panel (**C**) shows the difference in water temperature between the higher and lower temperature tank by temperature in the warmer tank. Panel (**D**) shows the same as panel (**C**) but with means calculated for temperature bins. The mean temperature difference between the warmer and cooler tanks was 1.3 °C (SD = 0.5 °C). Note that the blue, horizontal line indicates no preference (i.e., a y-intercept of 0.5).

The proportion of Delta Smelt in the cooler tank was influenced by temperature, as models that included temperature as a predictor received 99.57% of the AIC_c_ weight (Table 1). The top-ranked temperature model included the temperature of the warmer tank as a predictor, divided into three bins (Table 1; Fig. 2A and B). This model included a parameter for the low (9.1–13.2 °C), medium (14.0–28.0 °C), and high temperature (28.1–28.8 °C) bins. For the lowest temperature bin (< 14 °C), a higher percentage of Delta Smelt were in the warmer tank, with a model predicted percentage of fish in the cooler tank of 47.1%. However, this percentage did not differ significantly from 50% (95% CI 41.5, 52.5%), indicating no statistically significant preference for the warmer water. For the medium temperature bin (14–28 °C) there was a slight but statistically significant preference for the cooler tank, with a predicted percentage in the cooler tank of 55.1% (95% CI 51.9–58.4%). In the highest temperature bin (> 28 °C), Delta Smelt showed a strong preference for the lower temperature tank, with a predicted percentage in the cooler tank of 82.5% (95% CI 65.1, 96.7). While the second ranked model, which included acclimation temperature, received some AIC_c_ weight, the acclimation parameter was not significant (acclimation temperature parameter =  − 0.03, 95% CI − 0.11, 0.05; Table 1). We note that there was a parabolic relationship between the difference in temperature between the two tanks and water temperature (Fig. 2C and D). The temperature gradient was more difficult to maintain above and below ~ 18–20 °C, resulting in smaller differences between the tanks. Although a constant difference in temperature between the tanks would have been preferable, the temperature gradient was not a good predictor of Delta Smelt preference based on comparisons between Fig. 2B and C.

Although Delta Smelt demonstrated a strong preference for the cooler tank in the highest temperature bin, we observed increased mortality at 28 °C, regardless of acclimation temperature (14 or 17 °C; Fig. 2). No difference in behavior was observed up to 27 °C (aside from the slight preference for the cooler tank), whereas above 27 °C all individuals swam to the water surface and exhibited increased operculum movement and frequent gulping of air. The dissolved oxygen (DO) concentration in both chambers at the highest temperature pair (27.0 ± 0.1 °C and 29.4 ± 0.1 °C) was 7.45 ± 0.04 mg/L and 7.12 ± 0.03 mg/L, respectively, which represents 95.4% and 95.1% of oxygen saturation and was well within acceptable limits for the Delta Smelt.

### Salinity preference trials

**Figure 3:**
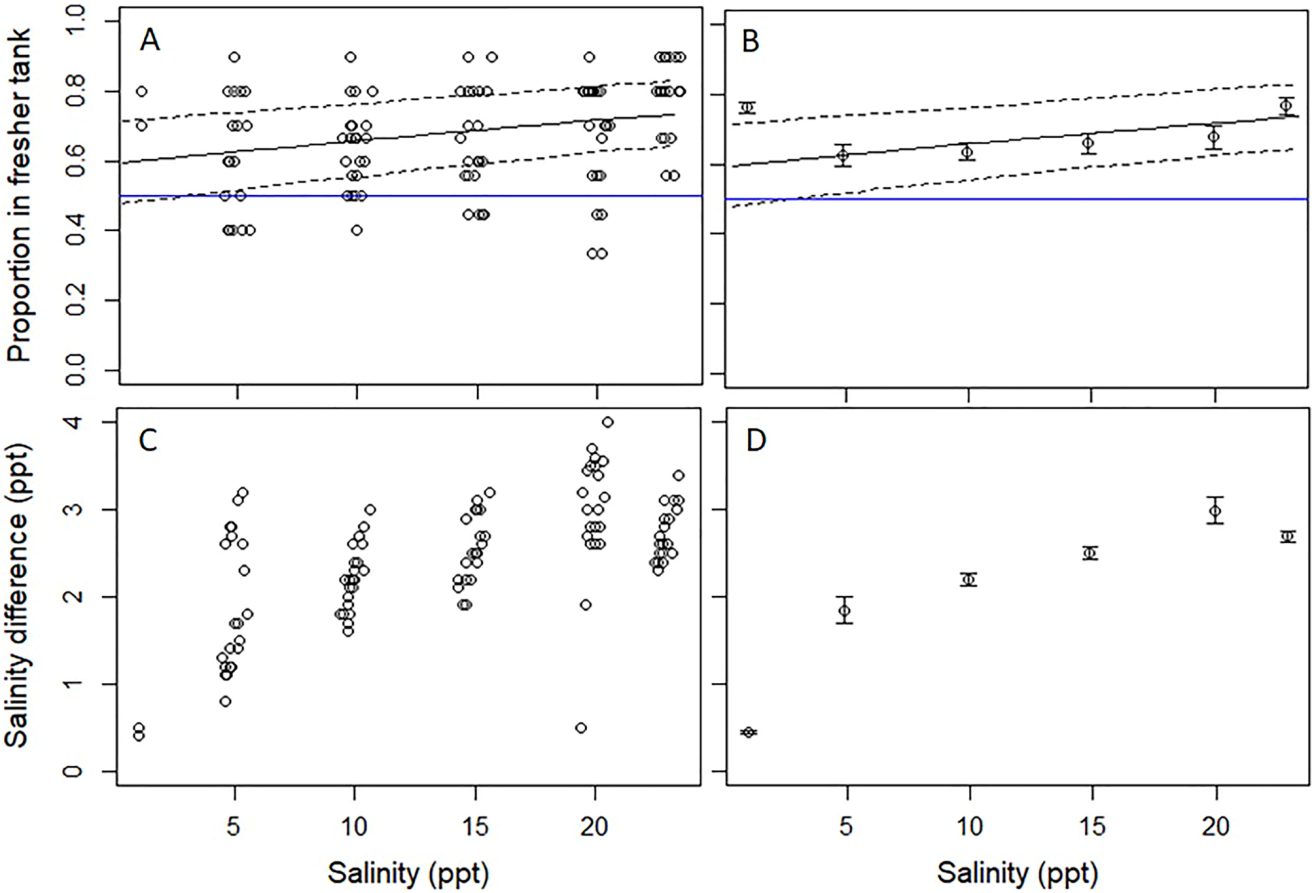
Panel (**A**) shows the proportion of sub-adult Delta Smelt in the fresher tank versus salinity in the higher salinity tank. The solid line represents the top-ranked (intercept model) and the dashed lines are the 95% CI. Panel (**B**) shows the same plot as panel (**A**) but with means calculated for temperature bins. Panel (**C**) shows a scatterplot of the difference in salinity between the two tanks by salinity in the higher salinity tank. Panel (**D**) shows the mean difference in salinity between the two tanks by salinity in the higher salinity tank. An average salinity difference of 2.2 ppt was maintained between the two tanks (SD = 0.9 ppt). Note that the blue, horizontal line indicates no preference (i.e., a y-intercept of 0.5).

In the salinity trials, the mean difference between the tanks was 2.2 ppt (SD = 0.9 ppt). The proportion of Delta Smelt in the fresher tank was influenced by salinity, as the top-ranked model included salinity (Table 2). In addition, Delta Smelt exhibited a statistically significant preference for the fresher tank (i.e., the confidence interval of the model did not include 0.5 across almost the entire salinity range; Fig. 3A and B, and the parameter estimate for salinity in the more saline tank was significant [0.03; 95% CI 0.01, 0.04]). This was likely driven by a stronger preference for fresher water by Delta Smelt at higher salinities rather than the larger difference in salinities between the tanks at higher salinities (Fig. 3C and D), as the model with salinity as a predictor outperformed the model with the difference in salinities between the tanks (i.e., Model 2 versus 3, respectively; Table 2). Based on model predictions, 59% of the fish preferred the fresher tank at the lower end of the salinity range (0.2 ppt), and 73% preferred the fresher tank at the higher end (22.0 ppt; Fig. 3).

**Table 2:** Model comparison for the salinity preference experiment. 'Sal' is the salinity of the more saline tank, ‘Sal difference’ is the difference in salinity between the two tanks, which was correlated with ‘Sal’ (i.e., the higher salinities had larger differences in salinity between the two tanks).

Model #	Model	ΔAIC_c_	df	AIC_c_ wt
2	~ Sal	0.0	3	0.937
3	~ Sal difference	6.2	3	0.041
1	~ Intercept	7.5	2	0.022

*ΔAIC*_*c*_: Difference between model of interest and top-ranked model in Akaike Information Criterion Units corrected for small sample size, *df*: Degrees of freedom, and *AIC*_*c*_* wt*: Akaike weight.

## Discussion

Animals live in complex environments where they modify their behavior to balance short-term survival with longer-term fitness^53–55^. The behavioral response depends on how the animal assesses a range of variables and how it reacts to maximize fitness. In the SFE, Delta Smelt catch decreases substantially in apparent response to increased salinities^20,22–24^ or temperatures^20,28,32–34^. It is unclear whether this is a behavioral or acute response, or a response to a covariable (e.g., predator or prey density). Our study evaluated the preferences of sub-adult Delta Smelt to a range of temperatures and salinities. Given the extreme drought conditions that California continues to endure, it is particularly important to consider how abiotic factors such as temperature and salinity play into Delta Smelt distribution, health, and fitness in the wild.

In our experiments, temperature preferences of sub-adult Delta Smelt were barely detectable across the vast majority of temperatures examined, with only a very modest preference for the cooler water across most of the temperature range. While the mean temperature gradient between the tanks was also fairly modest at 1.3 °C, teleosts can detect small temperature differences, including as small as 0.03 °C^56^. For example, Tuna (*Euthynnus affinis*) modify their behavior to temperature changes of 0.1 °C^57^. Thus, the temperature gradient should have been sufficient for a teleost like Delta Smelt to detect and respond to. A slight preference for cooler temperatures likely lowers metabolic demand of Delta Smelt, preserving energy in their food limited habitat^31^. At the highest temperatures, however, clear signs of stress were observed and the preference for the cooler tank was sharply delineated. Showing little to no influence of acclimation temperature was surprising, given the considerable research showing that increases in maximum thermal tolerance can be expected with higher acclimation temperatures^27–29,58^. For Delta Smelt, Komoroske et al. acclimated post-larval stages of Delta Smelt at target temperatures of 12 °C, 16 °C, and 19 °C, and found that while Delta Smelt acclimated at the lowest temperature had significantly reduced CT_max_ values compared to the medium- and high-temp acclimation fish, there was no difference in CT_max_ between the medium- and high-temp acclimation fish^21^. Possible explanations for the discrepancies are that our experiments evaluated the preferences rather than CT_max_ and subadult rather than juvenile Delta Smelt.

Swanson et al. reported that the critical thermal maximum for adult Delta Smelt is 25.4 °C^29^. However, in our study, and in Komoroske et al., the fish appeared calm at 25 °C, and only started to appear agitated when the temperature reached 27 °C^21^. Perhaps the most plausible explanation for the differences between Swanson et al.^29^ and more recent studies is that the Delta Smelt in our study and others^21,28,59^ were obtained from the hatchery where Delta Smelt are cultured (FCCL), whereas Swanson et al. used wild-caught Delta Smelt in their CTM experiments^29^. It is likely that the culture conditions at the FCCL are more optimal than those in the wild, which could account for the higher tolerance of the Delta Smelt we observed^60^. The wild-caught Delta Smelt underwent antibacterial and antifungal treatments during their acclimation period^29^, which also may have affected their temperature tolerance. In addition, cultured sub-adult Delta Smelt feeding on natural prey in unfiltered, untreated water pumped from the Delta also survived up to 27 °C^59^. Other explanations for the difference in CT_max_ values include a slower temperature adjustment during the preference trial period in the present study (6 h), a potential social component (possibly better performance in the presence of companions), or genetic differences in the test populations^61^. In any case, given that the current wild population appears to be at least partially comprised of hatchery raised Delta Smelt (or their offspring), our results are likely to be increasingly applicable to Delta Smelt in the wild.

Delta Smelt at the highest temperatures showed a strong preference for the lower temperature tank, yet we still observed signs of stress such as the increased flaring of operculum and gulping for air when fish were at the top of the water column^62^. As DO concentrations at 27 °C were well within the physiological range sufficient for Delta Smelt, a possible reason for them to stay near the water surface is that the water temperature at the surface was cooler than in the shuttlebox tanks (room temperature = 20 °C), which may have allowed the fish to regulate their body temperature. Heat conduction through the body surface in fish is a key mechanism in temperature regulation^63,64^. Moreover, fish have a highly specialized ability to differentiate temperature regimes and can behaviorally avoid conditions detrimental to their survival if a more suitable habitat is available^65–67^. Heat-induced hyperactivity assists with this escape behavior^27^.

In contrast to the temperature preference trials, Delta Smelt did not show signs of agitation or distress at high salinity. However, Delta Smelt showed a clear preference for the fresher water across a wide salinity range, and this preference increased in strength as salinity increased. Thus, our results indicate that Delta Smelt are able to tolerate a wide range of salinity despite their affinity for freshwater, which is consistent with the findings reported by others^21,29^. Consistent with our results, Delta Smelt distribution in the wild indicates a preference for lower salinity waters, especially during the summer where salinities are in the 1–2 ppt range^22,29,68^. Delta Smelt exhibit survival rates above 80% at 34 ppt in laboratory experiments, and show no differences in metabolic demand from freshwater to 12 ppt^25,69^. Thus, although the preference for fresher water provides a mechanism for why Delta Smelt distribution in the wild is restricted to the LSZ, it remains unclear why the species shows a preference for lower salinities. Our results are in-line with the assessment that Delta Smelt may be highly vulnerable to climate change^35^ and also indicated the conditions of SFE are becoming less favorable for the species. The findings in this study also provide valuable information in terms of Delta Smelt temperature and salinity preference to water managers and habitat restoration projects in the SFE to conserve the species.

## Conclusion

It is important to observe fish as they approach an environmental threshold to learn their behavioral response before conditions become acute. Movement away from an adverse condition is the behavioral response we attempted to capture here. Delta Smelt exhibited behavioral responses to both temperature and salinity, but with differing functional responses. The aversion of Delta Smelt to warm water was barely detectable over a wide range of temperatures, but became quite pronounced near the CT_max_ of the species (i.e., a nonlinear response to temperature). Results of the temperature preference study support the finding by Nobriga et al. that Delta Smelt capture probabilities decrease abruptly at temperatures higher than 24 °C in the SFE^33^ and suggest that the species is actively avoiding high temperatures in the wild. Our findings also suggest Delta Smelt will strongly avoid certain areas with sub-acute temperatures, such as some parts of Yolo Bypass floodway in summer^70^. We want to emphasize that our findings are most applicable to sub-adult Delta Smelt, as younger or older life-stages may have different preferences, as with temperature tolerances^21^. Like with temperature, the preference of Delta Smelt for fresher water became more pronounced as salinity increased, but strengthened linearly from freshwater to 23 ppt. Results of the salinity preference study align with Delta Smelt distribution in the wild, in that Delta Smelt actively seek out fresher water at the sub-adult stage, despite their ability to tolerate far higher salinities.

## Supplementary Information

Supplementary Information.

## Data Availability

Data are available upon request to the corresponding author.
