# Supplementary material for: Temperature and salinity preferences of endangered Delta Smelt (Hypomesus transpacificus, Actinopterygii, Osmeridae)
Source: Sci Rep. 2022 Oct 3;12:16558. doi: 10.1038/s41598-022-20934-w (PMC9530165; doi:10.1038/s41598-022-20934-w)
Supplement: Supplementary file 1 — Supplementary Information. [file 41598_2022_20934_MOESM1_ESM.docx]

**Supplemental Material —** Temperature and salinity preferences of endangered Delta Smelt (*Hypomesus transpacificus*, Actinopterygii, Osmeridae)

Tien-Chieh Hung^1,*^, Bruce G. Hammock^2^, Marade Sandford^1^, Marie Stillway^2^, Michael Park^2^, Joan C. Lindberg^1^, Swee J. Teh^2^

^1^Fish Conservation and Culture Laboratory

Department of Biological and Agricultural Engineering

University of California, Davis

Davis, CA 95616

^2^Aquatic Health Program

Veterinary Medicine: Anatomy, Physiology, and Cell Biology

University of California, Davis

Davis, CA 95616, USA

*Corresponding author: [thung@ucdavis.edu](mailto:thung@ucdavis.edu)

**Supplemental Figures**

(a)

(b)


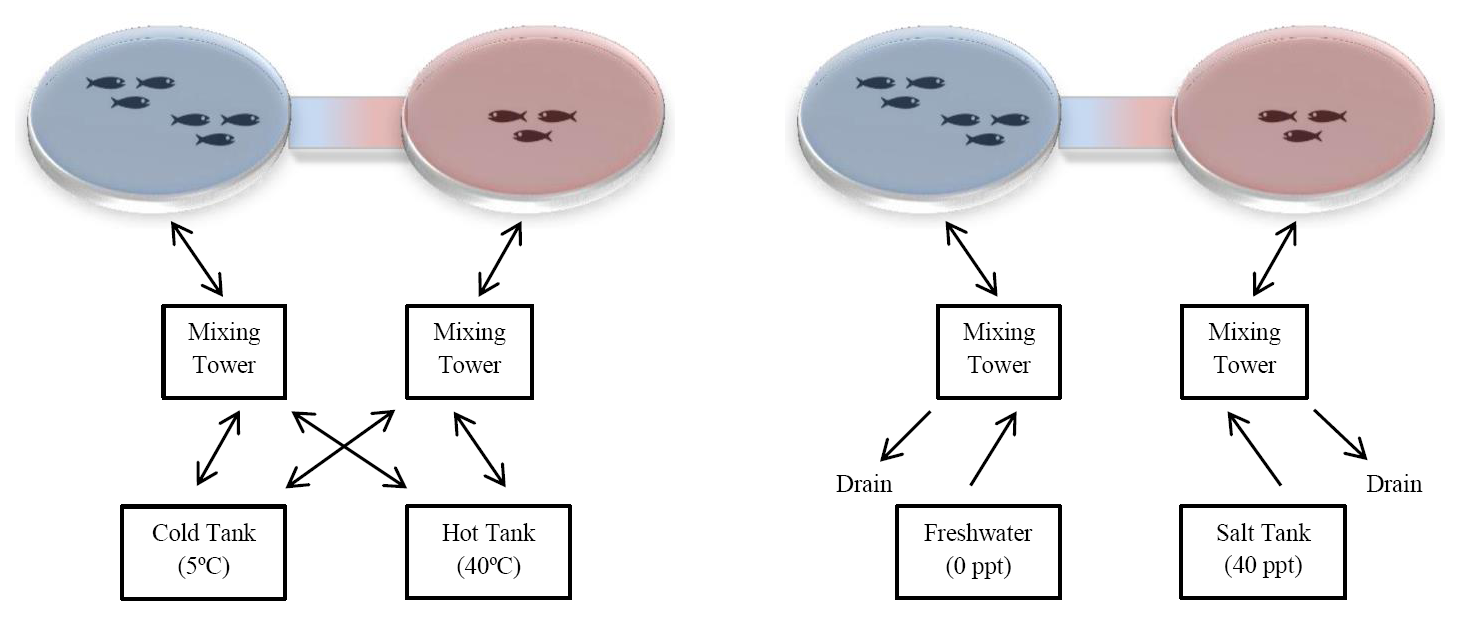


Figure S1. Schematic diagrams of the testing system for (a) temperature and (b) salinity trials.


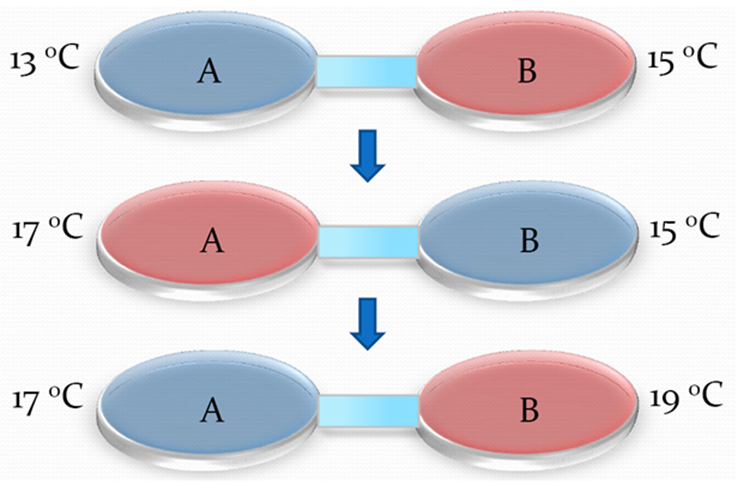


Figure S2. Operational strategy of temperature trials.


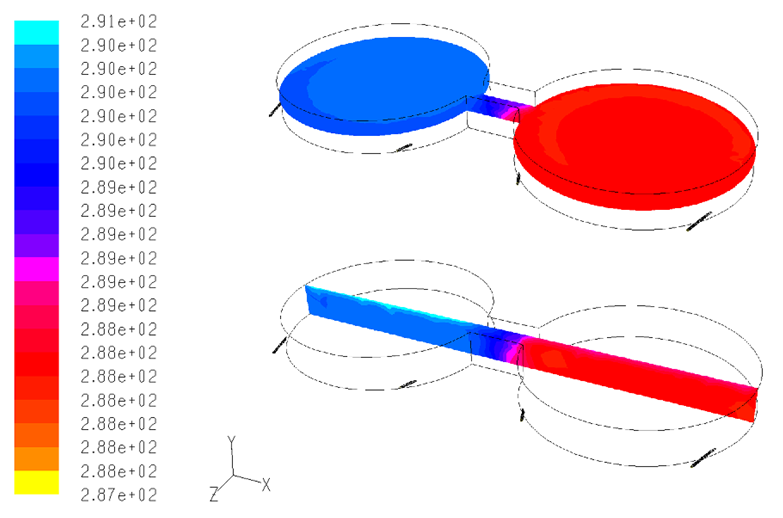


Figure S3. Temperature distribution at the middle of cross section of the testing system. The unit is Kelvin (K).
